# Age, gender, height and weight in relation to joint cartilage thickness among school-aged children from ultrasonographic measurement

**DOI:** 10.1186/s12969-021-00554-w

**Published:** 2021-05-12

**Authors:** Chun-Chun Gau, Tsung-Chieh Yao, Shu-Ting Gan, Syh-Jae Lin, Kuo-Wei Yeh, Li-Chen Chen, Liang- Shiou Ou, Wen-I Lee, Chao-Yi Wu, Jing-Long Huang

**Affiliations:** 1 Division of Allergy, Asthma, and Rheumatology, Department of Pediatrics, Chang Gung Memorial Hospital and Chang Gung University College of Medicine, Taoyuan, Taiwan; 2 Division of Pediatric General Medicine, Department of Pediatrics, Chang Gung Memorial Hospital at Keelung, Keelung, Taiwan; 3 Center for Big Data Analytics and Statistics, Chang Gung Memorial Hospital, Taoyuan, Taiwan; 4 Department of Pediatrics, New Taipei Municipal TuCheng Hospital, New Taipei city, Taiwan

**Keywords:** Pediatrics, Epidemiology, Ultrasonography, Cartilage thickness

## Abstract

**Background:**

Among school-age children, the decrease of cartilage thickness (Cth) with increasing age is well known. However, the influence of body mass index (BMI), height or weight on Cth has not been revealed. Here in, we aim to establish an age- and gender-specific Cth standard reference among Asians and investigate the possible prestige of BMI, height and weight.

**Methods:**

A cross-sectional study was performed in healthy Asian children. Bilateral knees, ankles, wrists, second metacarpophalangeals (MCPs) and proximal interphalangeals (PIPs) were measured using ultrasound. The children’s height, weight and BMI were also recorded for later adjustment.

**Results:**

A total of 200 school age Asian children (including 86 girls and 114 boys, aged between 5 to 13 years-old) were investigated. Cth differences were observed in the knees, ankles, wrists, MCPs and PIPs between sexes (*p* < 0.05), with girls having thinner cartilage thickness. While Cth decreases with increasing age (*p* < 0.0001, 0.039, 0.001, 0.023, 0.091 in girls’ knees, ankles, wrists, MCPs and PIPs and *p* = 0.002, 0.001, < 0.0001, 0.001, 0.045 in boys’, respectively). Our data showed that weight, height and BMI are not the main factors contributing to Cth. A formula to calculate gender-specific cartilage thickness for Asian school age children is suggested. There was no difference in Cth after adjusting for height or weight between Asian or Caucasian group.

**Conclusions:**

A formula to calculate gender-specific cartilage thickness for Asian school age children is suggested. Height, weight and BMI were not the major contributor for Cth among school age children.

**Supplementary Information:**

The online version contains supplementary material available at 10.1186/s12969-021-00554-w.

## Background

Musculoskeletal ultrasound examination technology has made great progress in recent decades, especially in the pediatric population. However, challenges in the growing skeleton, incomplete ossification and physiological vascularity among children have made sonography more difficult to interpretate [1–6].

Standard reference values provide the basis for pediatric musculoskeletal sonography had been established in a Danish study [7] and in India [8] focusing on Caucasians and multiethnic population. Their results indicated that with the growth of age, the thickness of hyaline cartilage may decline with decreasing cellular concentrate [9]. However, as the knee anthropometry had been revealed to be different between Caucasian and Asian via magnetic resonance imaging (MRI) survey [10], it is likely that Cth may vary among children with different ethnical background. In addition, discrepancies were found in the body mass index (BMI), body height and body weight according the the WHO standards and reference of Taiwan children [11]. Therefore, we aim to establish an age- and gender-specific Cth standard reference for school age children in Asia.

Moreover, along the advance of age, body height, weight and BMI also change simultaneously with time. Whether the decrease of cartilage thickness (Cth) is independently associated with age or is it also related to child’s body height and weight had not been previously investigated. Nonetheless, while childhood obesity becomes emerging problems worldwide [12], how obesity impact cartilage thickness also requires further survey.

Altogether, we aimed to determine the cartilage thickness in the healthy Asian children and investigate the contribution of height, weight and BMI in Cth.

## Methods

### Subjects

Two hundred healthy Taiwanese children aged 5 to 13 were recruited between January 2018 to June 2019, including 86 girls (mean age at 7.9 ± 2.0 years) and 114 boys (mean age at 8.3 ± 1.9 years). The distribution of age, gender, weight, height and BMI of the study subjects were listed in supplementary Table 1. Those with conditions which may potentially affect bone growth and metabolism, including the use of corticosteroid and growth hormone or a history of traumatic events and surgery of the joints were excluded. Children with chronic systemic diseases such as juvenile idiopathic arthritis, systemic lupus erythematous and other autoimmune/autoinflammatory diseases were also excluded. Simple physical examinations were performed to evaluate the joints for redness, swelling, tenderness and range of motion limitation. Date of birth, body height and weight were also recorded at the time. Body mass index was calculated by weight in kilograms divided by height in meters squared. Children were clustered into four groups (less than 25 percentile, 25 to 50 percentile, 50 to 75 percentile and more than 75 percentile) depending on their height, weight, BMI according to the 2010 growth charts for Taiwanese children and the World Health Organization Standard [11].

This cross-sectional study was approved by the Ethics Committee on Human Studies at Chang Gung Memorial Hospital in Taiwan, R.O.C. (IRB 201700405A3). Informed consents were signed by the children and at least one of their parents.

### Ultrasonography

Conventional B-mode on a real-time Direction Number 5460970–100 Revision 1, GE Healthcare LITEON eUAU108 with linear 12 L-RS 5.6 ± 20% MHz transducer were applied for the measurement. The cartilage thickness were measured according to the European League Against Rheumatism (EULAR) standard scan [13]. Specifically, the thickness of the white band was included into the Cth measured [14]. Three measurements were acquired from each joint and the mean was calculated to limit the measurement errors.

A pre-test was performed by three experienced pediatric rheumatologists, who had at least one-year experience on pediatric musculoskeletal sonography and performed exams on more than 100 cases, to minimize and validate the variability. Paired t-test was used to compare the intra-investigator variation. No significant difference were noted in all calculations (all *p* > 0.05). One principal investigator (CC Gau) performed the cartilage thickness measurements. The average observation time is about 30 min for each child.

### Joint measurement

#### Knee and ankle joints

The child was seated with knee maximally flexed and we took the cartilage thickness measurement from the suprapatellar transverse scan over the midline of the intercondylar notch. Next, the children’s foot was placed on the examination surface with knee flexion at 90 degrees to measure the tibiotalar joint cartilage thickness from the anterior longitudinal scan between first and second metatarsal bone. Specifically, Cth of talus was measured 5 mm from the dome of talus in the proximal direction and perpendicular over the bone surface.

#### Wrist and finger joints

In a seated position, children were asked to lay their hands at the pal-side position at the examination surface. We took the wrist cartilage thickness measurement on a dorsal longitudinal scan over the radial and scaphoid bones. After the measurement, we identified the cartilage thickness of dorsal second metacarpophalangeal (MCP) and proximal interphalangeal (PIP) joints with the joints flexed in 90 degrees under the transversal scan in the midline perpendicular in the bone surface.

### Statistical analysis

We used the linear regression and t-test to analysis the relations in age and cartilage thickness and clustered to boys and girls. Multiple joints cartilage thickness data were calculated with multiple linear regression models and clustered to four quartiles with comparison via Analysis of variance (ANOVA). Moreover, data were also analyzed with age, height and weight with multiple regression and compared to Denmark study with t-test [15]. Statistical analysis was performed with SAS 9.4, and a *p*-value less than 0.05 which was considered statistical significance.

## Results

A total of 200 pupils with total 2000 joints (bilateral knees, ankles, wrists, MCPs and PIPs) had been surveyed. No difference was observed between the right and the left joints evaluated. As shown in Table 1**,** girls’ cartilage thickness is significantly thinner than boys’. In addition, we found that the Cth of knees in girls decline faster than those in boys during school age (*p* = 0.031). The slope of Cth in the ankles, wrists, MCPs and PIPs were not statistically different between genders **(**Table 2**)**.

**Table 1:** Difference between girls and boys in cartilage thickness (0.1 mm), *p* < 0.05 for all joints

	Girls (*n* = 86) Mean ± SD (0.1 mm)	Boys (*n* = 114) Mean ± SD (0.1 mm)	*p*-value
Knee	33.6 ± 5.4	37.6 ± 5.3	<.0001*
Ankle	12.3 ± 3.4	13.8 ± 3.4	0.002*
Wrist	11.3 ± 2.8	12.2 ± 2.6	0.017*
MCP	7.3 ± 2.3	8.4 ± 2.0	0.001*
PIP	5.5 ± 1.6	5.9 ± 1.5	0.044*

*MCP* metacarpophalangeal joint, *PIP* proximal interphalangeal joint, *SD* standard deviation, * *p* < 0.05

**Table 2:** Difference between girls and boys decline in cartilage thickness (0.1 mm)

	Girls Boys Estimated slope	95%CI	SE	Boys estimated slope	95%CI		
Knee	−0.16	−0.20 to − 0.11	0.02	− 0.08	−0.13 to − 0.03	0.02	0.031*
Ankle	−0.04	−0.07 to 0.00	0.02	−0.05	− 0.08 to − 0.02	0.02	0.326
Wrist	−0.05	− 0.07 to − 0.02	0.01	−0.06	− 0.08 to − 0.04	0.01	0.272
MCP	−0.05	− 0.01 to 0.00	0.00	− 0.00	−0.01 to 0.00	0.00	0.376
PIP	−0.00	−0.00 to 0.00	0.00	−0.00	− 0.00 to 0.00	0.00	0.399

*MCP* metacarpophalangeal joint, *PIP* proximal interphalangeal joint, *SE* standard error, *CI* confidence interval, * *p* < 0.05

Body height, weight and BMI vary significantly with age [11]. To evaluate the potential impacts of these factors on Cth, we investigated the importance of height, weight and BMI in the joints with different prediction models by gender in Table 3. According to the data, we found that age, height, weight (model 1, 2 and 3) were significantly related to Cth individually but not BMI (model 4). Next, we adjusted the models based on the children’s age and height (model 5), age and weight (model 6) and all three variants (model 7). As shown in Table 3, multivariate models did not improve the fitness. The age alone model seems to outperform height, weight or BMI in predicting Cth. Nonetheless, we also divided height, BMI and weight into four groups to further analyze how theses parameters affect cartilage thickness. Demonstrated in Table 4, none of these parameters significantly influence the Cth in the 5 joints regardless of the children’s growth status.

**Table 3:** Different joint cartilage thickness after adjust age, height, weight and BMI statistics models

		Knee			Ankle			Wrist			MCP			PIP		
		beta	*p*-value	R^2^	beta	*p*-value	R^2^	Beta	*p*-value	R^2^	Beta	*p*-value	R^2^	beta	*p*-value	R^2^
Girls																
Model 1	Age	− 15.69	<.0001*	0.35	−3.67	0.039*	0.05	− 4.73	0.001*	0.12	−0.27	0.023*	0.06	−0.14	0.091	0.03
Model 2	Height	−0.07	0.008*	0.06	−0.05	0.056	0.04	−0.01	0.351	0.01	−0.12	0.001*	0.09	−0.20	<.0001*	0.27
Model 3	Weight	− 0.06	0.029*	0.04	−0.05	0.174	0.02	−0.02	0.258	0.02	−0.16	<.0001*	0.12	−0.24	<.0001*	0.21
Model 4	BMI	−0.11	0.086	0.01	−0.03	0.140	0.00	−0.07	0.065	0.01	−0.35	0.131	0.06	−0.51	0.218	0.06
Model 5	Age	− 21.51	0.001*	0.36	− 3.64	0.450	0.05	− 6.31	0.100	0.12	− 0.27	0.403	0.06	− 0.51	0.025*	0.07
	Height	0.90	0.327		0.00	0.995		0.25	0.655		0.00	0.998		0.06	0.081	
Model 6	Age	−18.48	<.0001*	0.36	−5.30	0.097	0.05	−5.99	0.019*	0.13	−0.12	0.580	0.07	−0.20	0.180	0.04
	Weight	0.67	0.426		0.39	0.535		0.30	0.545		−0.04	0.383		0.01	0.628	
Model 7	Age	−0.59	0.083	0.09	−0.38	0.433	0.06	−0.50	0.028*	0.07	−0.43	0.403	0.12	−2.16	0.001*	0.36
	Height	0.01	0.833		−0.03	0.678		0.07	0.078		0.04	0.663		0.07	0.518	
	Weight	0.00	0.970		0.06	0.457		−0.02	0.568		− 0.15	0.057		0.03	0.760	
Boys																
Model 1	Age	−8.00	0.002*	0.09	−5.17	0.001*	0.09	−6.28	<.0001*	0.23	−0.32	0.001*	0.10	−0.15	0.045*	0.04
Model 2	Height	−0.03	0.100	0.03	−0.02	0.243	0.01	−0.09	<.0001*	0.16	−0.07	0.001*	0.12	−0.06	<.0001*	0.11
Model 3	Weight	−0.05	0.040*	0.05	− 0.02	0.193	0.02	−0.08	0.001*	0.10	−0.08	0.006*	0.09	−0.07	<.0001*	0.12
Model 4	BMI	−0.20	0.100	0.04	−0.05	0.049*	0.01	−0.11	0.074	0.02	−0.16	0.119	0.02	−0.17	0.059	0.06
Model 5	Age	−3.76	0.472	0.09	−5.87	0.082	0.09	−6.18	0.009*	0.23	−0.13	0.504	0.11	−0.17	0.254	0.04
	Height	−0.73	0.356		0.12	0.811		−0.02	0.964		−0.03	0.275		0.00	0.830	
Model 6	Age	−2.69	0.446	0.12	−5.36	0.021*	0.09	−6.78	<.0001*	0.23	−0.19	0.160	0.11	−0.16	0.131	0.04
	Weight	−1.29	0.038*		0.05	0.907		0.12	0.660		−0.03	0.175		0.00	0.864	
Model 7	Age	−0.33	0.349	0.06	−0.17	0.401	0.02	−0.42	0.129	0.18	−0.50	0.214	0.14	0.01	0.981	0.13
	Height	0.06	0.347		0.02	0.608		−0.04	0.396		−0.02	0.802		− 0.03	0.492	
	Weight	−0.07	0.217		−0.02	0.523		0.01	0.738		0.02	0.754		−0.05	0.198	

*BMI* body mass index

* *p*-value < 0.05

**Table 4:** Different joint cartilages thickness (0.1 mm) categized into four quartiles by weight, height or BMI, SD: standard deviation (Group 1: less than 25 growth percentile, Group 2: 25 to 50 growth percentile, Group 3: 50 to 75 growth percentile, Group 4: more than 75 growth percentile)

Boys					
Height	Group 1 (*N* = 22)	Group 2 (*N* = 19)	Group 3 (*N* = 27)	Group 4 (*N* = 46)	*p*-value
	Mean ± SD	Mean ± SD	Mean ± SD	Mean ± SD	
Knee	37.1 ± 4.9	37.9 ± 4.1	38.3 ± 4.9	37.2 ± 6.2	0.371
Ankle	14.0 ± 3.1	14.4 ± 3.2	13.3 ± 3.1	13.8 ± 3.8	0.781
Wrist	12.1 ± 2.3	12.6 ± 2.2	11.8 ± 2.4	12.2 ± 2.9	0.923
MCP	8.9 ± 2.4	8.6 ± 1.9	8.0 ± 1.6	8.3 ± 2.1	0.774
PIP	5.7 ± 1.2	6.4 ± 1.9	5.9 ± 1.4	5.8 ± 1.5	0.523
BMI	Group 1 (*N* = 20)	Group 2 (*N* = 20)	Group 3 (*N* = 23)	Group 4 (*N* = 51)	*p*-value
	Mean ± SD	Mean ± SD	Mean ± SD	Mean ± SD	
Knee	38.2 ± 4.7	37.7 ± 4.6	39.8 ± 4.3	36.3 ± 5.9	0.435
Ankle	13.9 ± 2.8	14.1 ± 2.8	14.1 ± 3.5	13.6 ± 3.8	0.055
Wrist	11.4 ± 2.1	12.2 ± 2.1	13.3 ± 3.3	12.0 ± 2.4	0.871
MCP	8.6 ± 1.9	8.5 ± 2.0	8.7 ± 2.5	8.1 ± 1.9	0.100
PIP	5.6 ± 1.3	5.9 ± 1.9	6.3 ± 1.1	5.8 ± 1.6	0.545
Weight	Group 1 (*N* = 24)	Group 2 (*N* = 20)	Group 3 (*N* = 26)	Group 4 (*N* = 44)	*p*-value
	Mean ± SD	Mean ± SD	Mean ± SD	Mean ± SD	
Knee	38.4 ± 5.1	37.3 ± 4.2	38.8 ± 4.0	36.5 ± 6.4	0.775
Ankle	13.6 ± 3.0	14.7 ± 2.6	13.8 ± 3.3	13.6 ± 3.9	0.299
Wrist	11.8 ± 2.3	12.2 ± 2.1	12.4 ± 3.1	12.2 ± 2.6	0.323
MCP	8.8 ± 2.3	8.8 ± 2.1	8.3 ± 1.8	8.0 ± 1.9	0.882
PIP	5.7 ± 1.2	6.2 ± 1.9	5.9 ± 1.1	5.9 ± 1.6	0.276
Girls					
Height	Group 1 (*N* = 13)	Group 2 (*N* = 20)	Group 3 (*N* = 24)	Group 4 (*N* = 29)	*p*-value
	Mean ± SD	Mean ± SD	Mean ± SD	Mean ± SD	
Knee	33.1 ± 5.0	33.3 ± 4.4	34.3 ± 56.79	33.3 ± 6.2	0.050
Ankle	12.4 ± 4.1	11.8 ± 2.3	12.4 ± 30.77	12.6 ± 4.0	0.882
Wrist	11.0 ± 2.0	11.8 ± 3.2	11.1 ± 34.84	11.1 ± 2.2	0.698
MCP	7.1 ± 1.6	8.1 ± 3.6	6.9 ± 16.05	7.2 ± 1.8	0.774
PIP	4.9 ± 1.0	6.0 ± 1.8	4.9 ± 11.39	5.8 ± 1.8	0.369
BMI	Group 1 (*N* = 23)	Group 2 (*N* = 19)	Group 3 (*N* = 18)	Group 4 (*N* = 26)	*p*-value
	Mean ± SD	Mean ± SD	Mean ± SD	Mean ± SD	
Knee	34.2 ± 5.7	33.5 ± 5.0	32.4 ± 4.1	33.9 ± 6.4	0.903
Ankle	12.7 ± 2.9	11.2 ± 2.4	12.1 ± 4.0	13.0 ± 3.9	0.746
Wrist	11.2 ± 2.4	11.1 ± 2.1	11.3 ± 2.8	11.4 ± 3.6	0.891
MCP	8.0 ± 3.1	7.1 ± 1.6	7.3 ± 2.2	6.9 ± 1.7	0.987
PIP	5.4 ± 2.0	5.7 ± 1.7	5.5 ± 1.3	5.3 ± 1.4	0.356
Weight	Group 1 (*N* = 12)	Group 2 (*N* = 25)	Group 3 (*N* = 21)	Group 4 (*N* = 28)	*p*-value
	Mean ± SD	Mean ± SD	Mean ± SD	Mean ± SD	
Knee	34.3 ± 4.2	33.0 ± 5.8	34.7 ± 4.8	33.0 ± 6.1	0.820
Ankle	12.0 ± 2.5	12.6 ± 3.7	12.0 ± 2.6	12.5 ± 4.0	0.645
Wrist	11.6 ± 2.2	11.0 ± 2.1	11.4 ± 3.0	11.2 ± 3.4	0.659
MCP	8.3 ± 4.1	7.0 ± 1.2	7.5 ± 2.4	7.0 ± 1.8	0.959
PIP	5.2 ± 1.7	5.4 ± 1.3	5.7 ± 1.9	5.5 ± 1.6	0.340

**Fig. 1:**
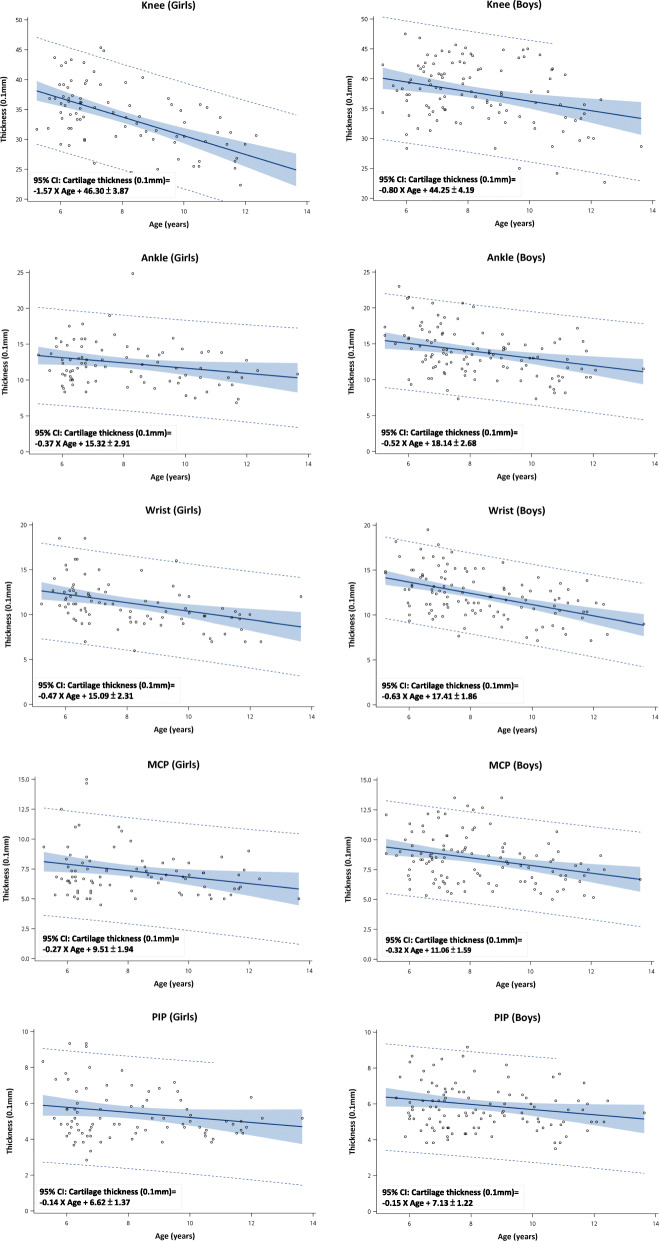
Measures of cartilage thickness in the knees, ankles, wrists, MCP and PIP joints of 5-13 elementary school boys and girls with mean (solid center line), 95% confidence interval (grey area) and predicted 95% confidence interval (dotted line)

As our data suggested that age and genders are the most important variables determining Cth among school age children, the measurement of each joints were depicted in Fig. 1 according to children’s gender and age with mean, 95% confidence interval(CI) and predicted 95% confidence interval. A formula calculating the Cth for the five evaluated joints within 95% confidence interval were also listed. In line with previous studies [7], a tendency of decreasing Cth along with the increase of age also be revealed in the Fig. 1.

Finally, we compared the Cth between Caucasian [7] and Asian children with estimated slope, 95% confidence interval and standard errors(SD) in the supplementary Table 2. The result suggested no differences in the Cth statically between Caucasian and Asian elementary-school age children regardless of their gender in the five joints evaluated. The compared *p*-value of boys’ and girls’ cartilage thickness over knees, ankles, wrists, MCPs and PIPS were 0.38 and 0.28, 0.36 and 0.38, 0.26 and 0.22, 0.06 and 0.06 and 0.09 and 0.08, respectively.

## Discussions

To the best of our knowledge, our study consisted of the first data analyzing the factors of children’s height, weight and BMI in association with Cth and we established the reference value of Cth for Asian school age children. In consistence with others [7], we found that the Cth in girls to be thinner than that of the boys. Besides, we demonstrated that age is the most critical factor in association with Cth in the school ages children as compared to body weight, height and BMI. Grouping children by their height, weight and BMI revealed no difference in the Cth of the evaluated joints. Finally, we established a standard reference of Cth in the knees, ankles, wrists, MCPs and PIPs for the Asian school age children and found that there were no significant differences between Caucasians and Asians.

In the present study, we discovered that the Cth were universally thicker in boys than girls among the evaluated joints. Moreover, the Cth of knees decline faster in girls than boys during their school age period [16]. Similar observation was also reported by Spannow A.H.et al in 2010 [7]. Among the 394 Danish children aged 7–16, Spannow discovered gender difference in Cth measurements and the steeper slope of Cth in the knee joints in girls. Although the exact mechanism remain to be clarified, estrogen receptors located on articular chondrocytes may likely play a role. The mean age at menarche was 11.35 years in Taiwan’s girl [17]. Estrogen has been shown to act on cartilage receptors and subchondral bone as a second messengers like regulatory polypeptides, similar to cartilage inducing factor alpha and transforming growth factor beta, to interfere cartilage turnover [18, 19]. Moreover, considering the differences in Cth between genders also existed in the prepuberty population, others and we hypothesized that physical activities and environmental factors may also contribute to the effects [19].

In line with Spannow and Moumita’s observation [7, 8], our data on school aged children between age 5 to 13 suggested that the Cth declines as the age advances. Although this linear correlation is true in our study as well as other reports [7, 8], it is not always the case since the measures of Cth in those preschool-aged-children and elder teens have been reported otherwise [20]. In the present study, without the very young children and older teens, we took advantage of the linear correlation and established a formula to calculate the Cth with 95% confident interval in the knees, ankles, wrists, MCPs and PIPs joints among Taiwanese school aged girls and boys. Worthwhile to say, however, extrapolation of our proposed formula in children outside of the targeted age may not be accurate. In adults, overweight people were found with thinner cartilage in their knees [21, 22]. Interestingly, Meng T et al. [23] following 186 participants from their childhood, discovered that the weight and BMI in the childhood were negative associated with the bone area and cartilage thickness in their knees after they reach adulthood. While Meng T assumed that between adolescence and adulthood, obesity affects different part of the knee joints (weight-bearing and non-weight-bearing), the association between body weight and Cth among school age children, however, has not been investigated yet. Moreover, the influence of body height on Cth was also surveyed for the very first time. With our extensive effects in evaluating these potential confounding factors, our data suggested that age is the leading contributor for Cth among school age children. Children with body weight or body height in different growth percentile do not have different Cth in the 5 joints evaluated.

In 2011, Yue B et al. reported that the elderly in China have smaller knees as compared to the Caucasians [10]. Nonetheless, among girls with a mean age of 11, Novotny R et al. discovered that girls with Asian ethnicity gained body size more slowly than those Caucasian girls without significant difference in the changes of the bone parameters [24]. In consistence with the observation, we also found no differences in the Cth among the five invested joints between the Asian children and the Caucasian population.

In this first study investigating the Cth in pure Asian children, adjusting for weight, height and BMI, our research has several limitations. One of these was the setting of a cross-sectional study in a single center setting. A multi-center research with serial follow up of the Cth from childhood into adolescence will provide a clearer picture of how Cth is affected by age. Another potential limitation of the present study is the inhomogeneous age distribution of study subjects. To establish the normal range of cartilage thickness in children of various age groups, a much larger sample size would have given more reliable results.

## Conclusions

We established a reference formula for the Cth in 5 investigated joints among school age Asian children and discovered that age and gender but not height, weight or BMI to be the major contributor for Cth among children within school age. Thus, a well-established Cth formula may be widely utilized by school-age children regardless of their body size and ethnicity.

## Supplementary Information

**Additional file 1: Supplement Table 1.** Characteristics of study participants by age (year).

**Additional file 2: Supplementary Table 2.** Differences in cartilage thickness between Danish and Asian children in schoolchildren age.

## Data Availability

All data generated or analyzed during this study are included in this article and its supplementary files.

## Abbreviations

Cth: Cartilage thickness
BMI: Body mass index MCPs Metacarpophalangeals
PIPs: Proximal interphalangeals
MRI: Magnetic resonance imaging
EULAR: European League Against Rheumatism
ANOVA: Analysis of variance
SD: Standard deviation
SE: Standard error
CI: Confidence interval
