# Supplementary material for: Age, gender, height and weight in relation to joint cartilage thickness among school-aged children from ultrasonographic measurement
Source: Pediatr Rheumatol Online J. 2021 May 12;19:71. doi: 10.1186/s12969-021-00554-w (PMC8117573; doi:10.1186/s12969-021-00554-w)
Supplement: Supplementary file 1 — Additional file 1: Supplement Table 1. Characteristics of study participants by age (year). [file 12969_2021_554_MOESM1_ESM.pdf]

Supplement Table 1. Characteristics of study participants by age (year)

Age	Gender Female (%)	Weight (Mean ± SD)	Height (Mean ± SD)	BMI (Mean ± SD)
5	50.0%	21.00±4.00	114.08±4.72	16.07±2.29
6	52.5%	21.33±3.74	116.94±5.61	15.55±2.16
7	26.5%	25.76±7.21	124.06±6.24	16.57±3.35
8	43.5%	26.33±4.39	128.51±5.38	15.91±2.00
9	41.7%	31.73±6.64	135.34±6.14	17.24±3.01
10	38.1%	40.22±10.31	141.62±6.46	19.96±4.49
11	44.4%	43.04±11.47	149.71±6.63	19.00±3.81
12	40.0%	45.50±9.44	152.54±7.99	19.39±2.41
13	50.0%	57.60±7.64	158.00±11.31	23.03±0.24

SD: standard deviation, BMI: body mass index
