# Supplementary material for: Age, gender, height and weight in relation to joint cartilage thickness among school-aged children from ultrasonographic measurement
Source: Pediatr Rheumatol Online J. 2021 May 12;19:71. doi: 10.1186/s12969-021-00554-w (PMC8117573; doi:10.1186/s12969-021-00554-w)
Supplement: Supplementary file 2 — Additional file 2: Supplementary Table 2. Differences in cartilage thickness between Danish and Asian children in schoolchildren age. [file 12969_2021_554_MOESM2_ESM.pdf]

Supplementary Table 2. Differences in cartilage thickness between Danish and Asian children in school-children age

	Spannow AH et al. (2010)				Our study				
	estimated slope	95%CI		SE	estimated slope	95%CI		SE	p-value
	boys (N=215)				boys (N=114)				
Knee	-0.07	-0.09	0.05	0.04	-0.08	-0.13	-0.03	0.02	0.388
Ankle	-0.04	-0.05	0.03	0.02	-0.05	-0.08	-0.02	0.02	0.360
Wrist	-0.12	-0.14	0.1	0.06	-0.06	-0.08	-0.04	0.01	0.260
MCP	-0.11	-0.12	0.1	0.06	0.00	-0.01	-0	0	0.064
PIP	-0.04	-0.05	0.04	0.02	0.00	0.00	-0	0	0.096
	girls (N=194)				girls (N=86)				
Knee	-0.11	-0.13	0.08	0.05	-0.16	-0.20	-0.11	0.02	0.288
Ankle	-0.03	-0.04	0.02	0.02	-0.04	-0.07	-0	0.02	0.383
Wrist	-0.11	-0.13	0.09	0.06	-0.05	-0.07	-0.02	0.01	0.220
MCP	-0.09	-0.1	0.08	0.05	0.00	-0.01	-0	0	0.064
PIP	-0.05	-0.06	0.05	0.03	0.00	0.00	0	0	0.088

CI: confidence interval, SE: standard errors
